# Antioxidant and antimalarial properties of *Sophora exigua* Craib. root extract in *Plasmodium berghei*-infected mice

**DOI:** 10.1186/s41182-021-00314-2

**Published:** 2021-03-19

**Authors:** Kantarakorn Kaewdana, Prapaporn Chaniad, Pitchanee Jariyapong, Arisara Phuwajaroanpong, Chuchard Punsawad

**Affiliations:** Department of Medical Sciences, School of Medicine, Walailak University, Nakhon Si Thammarat, 80160 Thailand

**Keywords:** Antimalarial activity, Antioxidant activity, Malaria, *Sophora exigua*

## Abstract

**Background:**

*Sophora exigua* Craib. is commonly used in Thailand to reduce fever and increase postpartum breast milk production in women who have hypogalactia. However, there has been no report on the antioxidant and antimalarial properties of this plant. This study aimed to investigate the antioxidant and antimalarial activities of *S. exigua* root extract and to evaluate its acute toxicity in mice to confirm its safety.

**Methods:**

The in vitro antioxidant activities were determined using 2,2-diphenyl-1-picrylhydrazyl (DPPH), superoxide radical, and hydroxyl radical scavenging assays. The in vivo antioxidant activities were determined by detecting the malondialdehyde (MDA) content and superoxide dismutase (SOD) activity in the livers of malaria-infected mice. The in vivo antimalarial activity was determined by Peters’ 4-day suppressive test in mice infected with *Plasmodium berghei* ANKA and orally administered *S. exigua* root aqueous and ethanolic extracts at different doses (200, 400, and 600 mg/kg body weight). In addition, the acute oral toxicity of the plant extracts was assessed in mice at a dose of 2000 mg/kg body weight.

**Results:**

The ethanolic extract of *S. exigua* root exhibited inhibition of DPPH radicals, superoxide anions, and hydroxyl radicals, with half maximal inhibitory concentration (IC_50_) values of 24.63 ± 1.78, 129.78 ± 0.65, and 30.58 ± 1.19 μg/ml, respectively. Similarly, research on the in vivo antioxidant activity indicated that the ethanolic extract of *S. exigua* root exerted a stronger effect than the aqueous extract. The aqueous extract at doses of 200, 400, and 600 mg/kg had stronger antimalarial activity than the ethanolic extract. The aqueous extract at 600 mg/kg exhibited 60.46% suppression of parasitemia. Increased levels of aspartate aminotransferase (AST), alanine aminotransferase (ALT), and alkaline phosphatase (ALP) and blood urea nitrogen (BUN) were detected in the mice treated with 2000 mg/kg ethanolic extract, which was related to the results of histopathological analysis of liver tissue, showing ballooning degeneration of hepatocytes, diffuse hepatic hemorrhage, and infiltration of inflammatory cells.

**Conclusions:**

This study demonstrated that the ethanolic *S. exigua* root extract possessed antioxidant properties, and the aqueous extract also had antimalarial activity. Therefore, this plant is an alternative source of new antioxidant and antimalarial agents.

## Introduction

Malaria is a disease caused by *Plasmodium* parasites, which infect humans and female *Anopheles* mosquitoes [1]. There are five species of *Plasmodium* that infect humans, including *Plasmodium falciparum* (*P. falciparum*), *Plasmodium vivax* (*P. vivax*), *Plasmodium ovale* (*P. ovale)*, *Plasmodium malariae* (*P. malariae*), and *Plasmodium knowlesi* (*P. knowlesi*) [1]. The World Malaria Report 2019 estimated that there had been 228 million cases of malaria worldwide, with 405,000 deaths from this disease. Among the deaths, children under 5 years of age constituted 272,000 cases [2]. Recent studies have suggested that the generation of reactive oxygen and nitrogen species (ROS and RNS) associated with oxidative stress plays a crucial role in the development of systemic complications caused by malaria [3]. The effects of ROS in malaria can be both beneficial and pathological, depending on the amount and site of production [4]. High production of free radical species such as nitric oxide that suppress the patient’s immune system has been related to malaria complications such as cerebral malaria [5] and acute kidney injury [6] and induces hepatic apoptosis [7].

The use of plant extracts and other natural products for antimalarial activity, antioxidant activity, and prevention of oxidative stress during malaria infection has been of interest for several years [3]. *Sophora* species have been studied for their antioxidant properties, and *S. flavescens* [8] and *S. japonica* [9] have been shown to be good candidates for antioxidants. In addition, (2*S*)-2′-methoxykurarinone, sophoraflavanone G, and leachianone A, isolated from the roots of *S. flavescens*, exhibited moderate antimalarial activity, with half maximal inhibitory concentration (IC_50_) values of 2.4, 2.6, and 2.1 μM, respectively [10]. Additionally, the other pharmacological effects of *Sophora* species include anticancer effects, induction of apoptosis, an effect on hair growth, and antimicrobial and antiviral activities [11].

*Sophora exigua* Craib., known locally in Thai as Phit sa nat, belongs to the Fabaceae family and is distributed in several parts of Thailand [11]. This plant is an ingredient of Kheaw-hom as traditional Thai remedy, which is commonly used for reducing fever and increasing breast milk production. It has been reported that an extract of *S. exigua* exhibited antioxidant [12] and antimicrobial activities against *Pseudomonas aeruginosa*, *Staphylococcus epidermidis*, and *Candida albicans* [13]. Additionally, sophoraflavanone G isolated from *S. exigua* exerts antibacterial effects by decreasing the fluidity of cellular membranes [14]. Based on previous studies, scientific data for *S. exigua* root extract have frequently demonstrated antimicrobial activity, but there is little information on its antioxidant activity and no report on its antimalarial activity. Therefore, this study aimed to investigate the antioxidant and antimalarial activities of *S. exigua* root extract and to evaluate the acute toxicity in mice to confirm the safety of the extract.

## Materials and methods

### Plant material and extraction process

The roots of *S. exigua* were obtained from a Thai traditional drug store in Nakhon Si Thammarat Province, Thailand, in 2019. The plant material was identified by Assoc. Prof. Tanomjit Supavita, School of Pharmacy, Walailak University, Thailand. The dried coarse powder of roots (60 g) was macerated three times with 95% ethanol (3 × 600 ml) at room temperature. The aqueous extract was extracted three times with 600 ml of distilled water under reflux for 3 h. The extract solutions were filtered with filter paper (Whatman no. 1) and evaporated under reduced pressure by a rotary evaporator (N-1200B, EYELA) to obtain aqueous (6.79 g) and ethanolic (4.9 g) extracts. Chromatographic fingerprint profiles obtained by a thin-layer chromatography (TLC) were generated for initial characterization of these extracts. The crude extracts were collected in sterile bottles and kept at 4 °C until use.

#### Phytochemical screening

The extracts of *S. exigua* roots were qualitatively screened to identify the presence of secondary metabolites, including flavonoids, terpenoids, alkaloids, tannins, steroids, cardiac glycosides, saponins, and coumarins [15].

### Evaluation of in vitro antioxidant activity

#### 2,2-Diphenyl-1-picrylhydrazyl (DPPH) radical scavenging activity assay

The free radical scavenging activity of crude plant extracts was measured by the 2,2-diphenyl-1-picrylhydrazyl (DPPH) assay [16] with some modifications. The plant extracts were diluted with dimethyl sulfoxide (DMSO) (Merck, Germany) to obtain twofold serial dilutions (1.562–400 μg/ml). The reaction mixture, containing 75 μl of plant extract and 150 μl of DPPH radical solution (0.1 mM in absolute ethanol), was added into a 96-well plate and incubated in the dark at room temperature for 30 min. L-ascorbic acid (1.562–100 μg/ml) was used as a positive control. The absorbance was measured at 517 nm by using a microplate reader. The assays were performed in triplicate. The percentage of the radical scavenging activity (%RSA) was calculated by using the formula below:

1 $$ \%\mathrm{RSA}=\frac{\mathrm{Abs}\ \mathrm{control}-\mathrm{Abs}\ \mathrm{sample}}{\mathrm{Abs}\ \mathrm{control}}\times 100 $$

where Abs control is the absorbance of DPPH radical + ethanol and Abs sample is the absorbance of DPPH radical + plant extract.

#### Superoxide anion scavenging assay

The superoxide anion scavenging activity was measured as described previously with some modifications [16]. Briefly, the plant extracts were diluted with DMSO to obtain twofold serial dilutions (1.562–400 μg/ml). The reagent mixture, containing 50 μl of 0.2 mM nitro blue tetrazolium chloride (NBT) solution (Merck, Germany), 50 μl of 25 μM phenazine methosulfate (PMS) solution (Merck, Germany), 50 μl of 0.5 mM nicotinamide adenine dinucleotide (NADH) solution (Sigma-Aldrich, USA) and 100 μl of plant extract, was added into 96-well plates. L-ascorbic acid (Ajax Finechem, USA) was used as a positive control. The plate was incubated at room temperature for 10 min, and the absorbance was measured at 560 nm. The assays were performed in triplicate. The percentage of superoxide radical scavenging activity (SRSA) of the plant extract was calculated by using the formula below, and the results are presented as IC_50_ values.

2 $$ \%\mathrm{SRSA}=\frac{\mathrm{Abs}\ \mathrm{control}-\mathrm{Abs}\ \mathrm{sample}}{\mathrm{Abs}\ \mathrm{control}}\times 100 $$

where Abs control is the absorbance without sample and Abs sample is the absorbance with sample.

#### Hydroxyl radical scavenging assay

Hydroxyl radical scavenging activity (HRSA) was determined according to the method of Halliwell and Gutteridge [17]. The reaction mixture consisted of 100 μl of 50 mM 2-deoxyribose (Sigma-Aldrich, USA), 100 μl of 50 mM hydrogen peroxide (Merck, Germany), 100 μl of 3.2 mM iron chloride (Sigma-Aldrich, USA), and 100 μl of 1 mM disodium EDTA (Sigma-Aldrich, USA), with or without 100 μl of plant extract at various concentrations. Butylated hydroxytoluene (BHT) (Sigma-Aldrich, USA) was used as a positive control. The reaction was triggered by adding 100 μl of 1.8 mM L-ascorbic acid (Ajax Finechem, USA) and incubated at 37 °C for 60 min. The reagent mixture, containing 500 μl of 10% trichloroacetic acid (TCA) (Merck, Germany) and 500 μl of 5% 2-thiobarbituric acid (TBA) (Merck, Germany), was added and boiled in a water bath at 95 °C for 30 min. After cooling at room temperature for 10 min, the absorbance was measured at 532 nm. The assays were performed in triplicate. The percentage of HRSA was calculated by using the following formula, and the results are shown as IC_50_ values:

3 $$ \%\mathrm{HRSA}=\frac{\mathrm{Abs}\ \mathrm{control}-\mathrm{Abs}\ \mathrm{sample}}{\mathrm{Abs}\ \mathrm{control}}\times 100 $$

where Abs control is the absorbance without sample and Abs sample is the absorbance with sample.

#### Experimental animals

Male ICR mice weighing 24 to 30 g and aged 4 to 6 weeks were purchased from Nomura Siam International Co., Ltd., Bangkok, Thailand. After the mice arrived at the Animal Research Center, Walailak University, all of them were kept in plastic cages for 7 days before starting the experiment. All experimental animals were housed under standard environmental conditions at a temperature of 22–24 °C under a 12-h light/dark cycle and had free access to standard pellets and water.

#### Malaria parasite inoculation

For thawing of malaria parasites, a frozen vial was placed in a 37 °C water bath until completely thawed. The malaria parasites were transferred aseptically into a sterile syringe. For inoculation, 200 μl of *P. berghei* ANKA-infected red blood cells were injected intraperitoneally into the donor mice. The malaria parasites were obtained through BEI Resources, NIAID, NIH: *Plasmodium berghei*, Strain ANKA, MRA-311, contributed by Thomas F. McCutchan. When the percentage of parasitemia increased to 5-10%, the donor mice were anesthetized with 60 mg/kg body weight sodium pentobarbital (Nembutal; Ceva, France), and the blood was collected via cardiac puncture into a heparinized vacutainer tube and used to induce malaria infection in the naive mice for the 4-day suppressive test.

#### Four-day suppressive test

The 4-day suppressive test was used to measure the schizonticidal activity of the aqueous and ethanolic extracts against *P. berghei*-infected ICR mice, following a previously described method [18]. Male ICR mice were randomly divided into 9 groups of 5 mice each, including the uninfected group, infected untreated group, artesunate group and six experimental groups with different doses (200, 400 and 600 mg/kg) of aqueous or ethanolic extract. The mice were injected intraperitoneally with the blood containing 1 × 10^7^ parasitized red blood cells of *P. berghei* ANKA [19]. The six treatment groups were administered aqueous or ethanolic extract of *S. exigua* orally at daily doses of 200, 400 and 600 mg/kg. The infected untreated group received 200 μl of the solvent used to dissolve the extracts, whereas the positive control group was orally administered 6 mg/kg body weight artesunate (Sigma-Aldrich, USA) per day. Treatment started 3 h after the mice had been inoculated with the parasite on day 1 and then continued daily for 4 days (that is, from day 1 to day 5). On day 5, Wright-Giemsa-stained thin blood smears were prepared from the tail of each animal to detect parasitemia, and percent suppression (% suppression) was calculated by using the formula below:

4 $$ \%\mathrm{Suppression}=\frac{\left[\mathrm{A}-\mathrm{B}\right]}{\mathrm{A}}\times 100 $$

where *A* is the average percentage of parasitemia in the infected untreated group and *B* is the average percentage of parasitemia in the extract-treated group.

### Evaluation of in vivo antioxidant activity

#### Measurement of malondialdehyde

Malondialdehyde (MDA) was measured by using a commercial kit (cat no. MAK085, Sigma-Aldrich, USA) according to the manufacturer’s instructions. Briefly, liver tissue (50 mg) was homogenized on ice in 300 μl of MDA lysis buffer containing 3 μl of BHT (100×) and centrifuged at 10,000×*g* for 10 min to remove insoluble material. The supernatant was collected in a microcentrifuge tube. Then, 0.1 M MDA standard solution was prepared by diluting 10 μl of 4.17 M MDA standard solution with 407 μl of water. To prepare a 2 mM MDA standard, 20 μl of the 0.1 M MDA standard solution was diluted with 980 μl of water. Then, 0, 2, 4, 6, 8 and 10 μl of the 2 mM MDA standard was placed in microcentrifuge tubes. Distilled water was added to each microcentrifuge tube to obtain a final volume of 200 μl and to generate 0 (blank), 0.4, 0.8, 1.2, 1.6 and 2.0 nM standards. A total of 600 μl of the TBA solution was mixed into each microcentrifuge tube containing sample or standard solution and incubated at 95 °C for 60 min. After cooling in an ice bath for 10 min, 200 μl of solution was pipetted from the microcentrifuge tube into a 96-well plate. The assays were performed in triplicate. The absorbance was measured at 532 nm, and the level of MDA was calculated by the following formula:

5 $$ \mathrm{C}=\left({\mathrm{S}}_{\mathrm{a}}/{\mathrm{S}}_{\mathrm{v}}\right)\times \mathrm{D} $$

where *C* is the concentration of MDA in sample, *S*_*a*_ is the amount of MDA in unknown sample (nmol) from standard curve, *S*_*v*_ is the sample volume (ml) or amount (mg) added into the wells and *D* is the sample dilution factor (if applicable).

#### Measurement of superoxide dismutase activity

Superoxide dismutase (SOD) activity was measured by using a commercial kit based on the WST-8 method (cat no. 19160, Sigma-Aldrich, USA). Briefly, 50 mg of liver tissue was homogenized on ice in cell lysis buffer (Cell Signaling Technology, USA) and centrifuged at 10,000×*g* for 10 min to remove insoluble material. The supernatant was collected into a microcentrifuge tube. Twenty microliters of sample was added to each sample and the blank 2 well. Twenty microliters of ultrapure H_2_O was added to the blank 1 and blank 3 wells. Two hundred microliters of WST working solution was added to each well, and 20 μl of dilution buffer was added to each blank 2 and blank 3 well. Finally, 20 μl of enzyme working solution was added to each sample and blank 1 well and incubated at 37 °C for 20 min. All tests were performed in triplicate. The absorbance was measured at 450 nm by using a microplate reader. SOD inhibition was calculated by using the following equation:

6 $$ \mathrm{SOD}\;\mathrm{inhibition}\;\left(\%\right)=\left({\mathrm{A}}_{\mathrm{Blank}\kern0.17em \mathrm{control}\;1}\hbox{-} {\mathrm{A}}_{\mathrm{Standard}\kern0.17em \mathrm{or}\ \mathrm{sample}}\right)/\left({\mathrm{A}}_{\mathrm{Blank}\kern0.17em \mathrm{control}\kern0.24em 1}\hbox{-} {\mathrm{A}}_{\mathrm{Blank}\ \mathrm{control}\;2}\right)\times 100\% $$

#### Acute toxicity test

The protocol for the acute toxicity test followed the OECD guidelines (2008) [20]. Twenty mice were randomly divided into 4 groups of five mice each. Group I received normal food and water intake without any treatment (untreated group). Group II mice were treated with 200 μl of 7% Tween 80 and 3% ethanol solution, serving as the negative control group, and groups III and IV were treated with 2000 mg/kg aqueous and ethanolic extracts, respectively. After treatment, the mice were continuously observed for changes in physical and behavioral activities for 1 h. In addition, the mice were observed daily for 14 days for clinical symptoms and behavioral changes, including central nervous, cardiovascular and gastrointestinal system symptoms, body weight changes, and changes in water and food consumption. On day 14, the mice were anesthetized with 60 mg/kg sodium pentobarbital (Nembutal; Ceva, France). The blood was harvested via the cardiac puncture technique for liver and kidney function tests. In addition, liver and kidney tissues were collected and preserved in 10% formaldehyde for histopathological analysis.

#### Biochemical analysis

At the end of the acute toxicity test, the blood was centrifuged at 3000xg for 10 minutes to prepare plasma samples. The plasma samples were transferred to the Medical Technology Clinic, Walailak University, for measurement of liver function (aspartate aminotransferase (AST), alanine aminotransferase (ALT) and alkaline phosphatase (ALP)) and kidney function (blood urea nitrogen (BUN) and creatinine (Cr)) by using an AU480 Chemistry Analyzer (Beckman Coulter, Brea, CA, USA).

#### Histopathological examination

The liver and kidney tissues were collected and fixed with 10% formaldehyde for 24–48 h and transferred to the Diagnostic Pathology Unit, Department of Tropical Pathology, Mahidol University, for tissue processing and staining. The tissues were dehydrated with an ethanol gradient, incubated in xylene and embedded in paraffin blocks. The paraffin blocks were sectioned at a thickness of 5 μm using a microtome and stained with hematoxylin and eosin (H&E). To determine histopathological changes, the stained sections were observed under a light microscope by two independent observers who were blinded to the experimental groups.

#### Statistical analysis

Statistical analysis was performed using SPSS for Microsoft Windows, release 17.0 (SPSS, IL, USA). The data was normally distributed assessed by the Shapiro-Wilk test. The differences in parameters among groups were tested using one-way ANOVA, followed by post hoc Tukey’s multiple comparison test. The level of statistical significance (*p* value) used was less than 0.05 (*p* < 0.05).

## Results

### Phytochemical screening

The phytochemical screening of the *S. exigua* root ethanolic extract revealed the presence of high levels of flavonoids, including terpenoids, saponins and coumarins, whereas the *S. exigua* root aqueous extract contained high levels of alkaloids with low levels of flavonoids and terpenoids (Table 1).

**Table 1:** Phytochemical screening of *S. exigua* root extract

Phytochemical constituent	Type of extract	
	Ethanol	Aqueous
Flavonoids	+++	+
Terpenoids	+	+
Alkaloids	−	+++
Tannins	−	−
Steroids	−	−
Cardiac glycosides	−	−
Saponins	++	−
Coumarins	+	−

+++ represents a strong presence; ++ represents moderate presence; + represents slight presence; − represents absence

### In vitro antioxidant properties of *S. exigua* root extract

The antioxidant property was expressed as the IC_50_ value. The DPPH radical assay determined the IC_50_ values of the ethanolic extract, aqueous extract and positive control as 24.63 ± 1.78, 116.32 ± 4.14 and 3.99 ± 0.26 μg/ml, respectively. The SRSA assay determined the IC_50_ values of the ethanolic extract, aqueous extract and positive control as 129.78 ± 0.65, 184.62 ± 3.13 and 11.77 ± 0.16 μg/ml, respectively (Table 2). The HRSA assay determined that the IC_50_ values of the ethanolic extract, aqueous extract and positive control were 30.58 ± 1.19, 44.04 ± 1.37 and 4.06 ± 0.95 μg/ml, respectively (Table 2). A lower IC_50_ value indicated a stronger antioxidant effect of the sample. The results from the three methods revealed that the IC_50_ of the ethanolic extract was lower than the IC_50_ value of the aqueous extract. Therefore, the in vitro antioxidant study indicated that the ethanolic extract of *S. exigua* roots had stronger antioxidant activity than the aqueous extract.

**Table 2:** In vitro antioxidant properties of L-ascorbic acid (a positive control), aqueous and ethanolic extracts of *S. exigua* roots

Samples	IC_**50**_ (μg/ml) for radical scavenging		
	DPPH radical	Superoxide radical	Hydroxyl radical
Ethanolic extract	24.63 ± 1.78	129.78 ± 0.65	30.58 ± 1.19
Aqueous extract	116.32 ± 4.14	184.62 ± 3.13	44.04 ± 1.37
L-ascorbic acid	3.99 ± 0.26	11.77 ± 0.16	4.06 ± 0.95

### In vivo antioxidant properties of *S. exigua* root extract

#### Measurement of MDA level

At the end of the 4-day suppressive test, liver homogenates from mice were used to evaluate the levels of MDA using a lipid peroxidation assay kit. In the present study, malaria-infected mice exhibited a significant increase in MDA levels in liver homogenate compared to the infected untreated group (Table 3). Malaria-infected mice treated with ethanolic extract at doses of 200, 400 and 600 mg/kg had significantly reduced MDA levels compared to the infected untreated group (*p* < 0.05), whereas aqueous extract at doses of 400 and 600 mg/kg significantly decreased the MDA level in malaria-infected mice compared with that in the infected untreated group (*p* < 0.05). In this study, the antioxidant activity of the ethanolic extract was higher than that of the aqueous extract (Table 3).

**Table 3:** In vivo antioxidant properties of the aqueous and ethanolic extracts during *P. berghei* ANKA infection

Group	MDA level (nmol/mg)	SOD inhibition (%)
Uninfected group	6.55 ± 1.11^a^	75.05 ± 3.53^a^
Infected untreated group	18.38 ± 1.34	57.89 ± 1.05
Artesunate 6 mg/kg	7.42 ± 1.61^a^	73.07 ± 0.02^a^
Ethanolic extract 200 mg/kg	8.58 ± 0.90^a^	61.60 ± 3.92
Ethanolic extract 400 mg/kg	8.41 ± 0.70^a^	64.82 ± 3.00^a^
Ethanolic extract 600 mg/kg	7.36 ± 0.84^a^	67.12 ± 2.34^a^
Aqueous extract 200 mg/kg	16.65 ± 1.86	59.86 ± 0.61
Aqueous extract 400 mg/kg	10.27 ± 0.67^a^	63.79 ± 1.74^a^
Aqueous extract 600 mg/kg	8.95 ± 0.75^a^	64.82 ± 3.00^a^

Data are presented as the mean ± SD (*n* = 5 per group)

Data were analyzed by one-way ANOVA, followed by post hoc Tukey’s multiple comparison test

^a^*p* < 0.05, significantly different compared to the infected untreated group

#### Measurement of SOD activity

SOD antioxidant enzyme activity was measured in tissue homogenate by using an SOD activity determination kit, and the results are shown in Table 3. The mean percentage of SOD inhibition was higher in the tissue homogenate of malaria-infected mice treated with ethanolic and aqueous extracts at doses of 200, 400 and 600 mg/kg than in the malaria-infected group (*p* < 0.05). In addition, this study found that malaria-infected mice treated with artesunate (6 mg/kg, as a positive control) exhibited higher inhibition than nontreated malaria-infected mice (*p* < 0.05). The malaria-infected mice treated with ethanolic extract showed the highest inhibition, with a value of 67.12 ± 2.34 at a dose of 600 mg/kg. In this study, the ethanolic extract also showed higher antioxidant activity than the aqueous extract.

#### Antimalarial activity of *S. exigua* root extract

The percentage of parasite suppression in each group was calculated to determine the antimalarial effect and is summarized in Table 4. This study indicated that ethanolic and aqueous extracts showed dose-dependent chemosuppressive activity against *P. berghei* ANKA. The percent suppression by the ethanolic extract of the plant was 29.60, 47.47 and 53.09% at doses of 200, 400 and 600 mg/kg, respectively. The percent suppression by the aqueous extract at doses of 200, 400 and 600 mg/kg was 36.64, 52.16 and 60.46%, respectively. The ethanolic and aqueous extracts showed more effective parasite suppression at the highest dose of 600 mg/kg than at 200 and 400 mg/kg (*p* < 0.05). Comparison of the effects of the ethanolic and aqueous *S. exigua* root extracts at the same dosage revealed that the aqueous extract showed stronger parasite suppression than the ethanolic extract at all dosages (*p* < 0.05).

**Table 4:** Antimalarial activity of artesunate (a positive control), aqueous and ethanolic extracts

Group	% Parasitemia	% Suppression
Infected untreated group	6.57 ± 1.89	**-**
Artesunate 6 mg/kg	0.18 ± 0.56	97.15 ± 0.92
Ethanolic extract 200 mg/kg	5.17 ± 0.89	29.60 ± 3.96 ^a,b^
Ethanolic extract 400 mg/kg	4.62 ± 0.34	47.47 ± 0.38 ^a,c^
Ethanolic extract 600 mg/kg	3.45 ± 0.03	53.09 ± 0.64 ^c^
Aqueous extract 200 mg/kg	4.16 ± 0.33	36.64 ± 4.21^c^
Aqueous extract 400 mg/kg	3.14 ± 0.35	52.16 ± 3.77 ^c^
Aqueous extract 600 mg/kg	2.59 ± 0.03	60.46 ± 0.38

Data are presented as the mean ± SD (*n* = 5 per group)

Data were analyzed by one-way ANOVA, followed by post hoc Tukey’s multiple comparison test

^a^*p* < 0.05, significantly different compared to 600 mg/kg ethanolic extract

^b^*p* < 0.05, significantly different compared to 200 mg/kg aqueous extract

^c^*p* < 0.05, significantly different compared to 600 mg/kg aqueous extract

### Acute toxicity test of *S. exigua* root extract

#### Monitoring general behavioral changes and percentage body weight changes during the acute toxicity test

Untreated group and the aqueous extract group exhibited no gross physical and behavioral changes. The ethanolic extract group exhibited decreased locomotor activity and increased self-grooming and hand licking activities. The body weights of the mice were measured at day 0 and day 14 to analyze the percentage of body weight change in each group. The percentage of body weight change was 4.44 in the aqueous extract group and 2.68 in the ethanolic extract group (Table 5). The percentage of body weight change of mice treated with plant extract was significantly lower than that of the untreated group (*p* < 0.05).

**Table 5:** Evaluation of percent body weight change in the untreated, aqueous and ethanolic extract groups during acute toxicity experiments

Groups	Mean body weight (g)		% Change
	Day 0	Day 14	
Untreated group	31.63 ± 0.30	35.39 ± 0.83	11.88 ± 3.71
Aqueous extract 2000 mg/kg	26.59 ± 0.99	27.76 ± 0.86	4.44 ± 1.59^a^
Ethanol extract 2000 mg/kg	27.26 ± 1.60	27.98 ± 1.33	2.68 ± 2.21^a^

Data are presented as the mean ± SD (*n* = 5 per group)

Data were analyzed by one-way ANOVA, followed by post hoc Tukey’s multiple comparison test

^a^*p* < 0.05, significantly different compared to the untreated group

#### Evaluation of the effect of *S. exigua* root extract on food and water intake

Water intake and food consumption were measured every day during the acute toxicity test. The water intake and food consumption data were separately analyzed for the first and second weeks and are displayed in Table 6. In the first week, there were no significant differences in water intake and food consumption between the extract-treated groups. In the second week, water intake and food consumption were significantly reduced in mice that received ethanolic extract at a dose of 2000 mg/kg compared with the untreated group and aqueous extract group (*p* < 0.05).

**Table 6:** Daily monitoring of food and water consumption in the untreated, aqueous and ethanolic extract groups during acute toxicity experiments

Parameter	Group		Ethanolic extract
	Untreated	Aqueous extract	
Food consumption (g/week)			
Week 1: Days 1–7	27.85 ± 3.93	26.42 ± 6.26	26.85 ± 3.76
Week 2: Days 8–14	23.57 ± 4.75	26.14 ± 7.35	19.28 ± 3.45^a,b^
Water consumption (ml/week)			
Week 1: Days 1–7	25.71 ± 7.31	27.42 ± 3.73	22.85 ± 5.66
Week 2: Days 8–14	23.57 ± 3.77	20.71 ± 3.45	17.21 ± 3.78^a,b^

Data are the mean ± SD (*n* = 5 per group)

Data were analyzed by one-way ANOVA, followed by post hoc Tukey’s multiple comparison test

^a^*p* < 0.05, significantly different compared to the untreated group

^b^*p* < 0.05, significantly different compared to the aqueous extract group

#### Biochemical parameters

Biochemical parameters for liver and kidney functions were analyzed to determine the toxicity of *S. exigua* root extract as shown in Table 7. In the analysis of liver function, the plasma level of AST was significantly elevated in the mice treated with aqueous and ethanolic extracts compared with the untreated group (*p* < 0.05). The plasma level of ALT and ALP was significantly increased in the mice treated with ethanolic extract compared to the untreated group, negative control and aqueous extract group (*p* < 0.05). These findings indicated that ethanolic extract at a dose of 2000 mg/kg induced liver toxicity. In the kidney function tests, the level of BUN was significantly increased in the mice treated with ethanolic extract compared to the untreated group, negative control group and aqueous extract group (*p* < 0.05). On the other hand, there was no significant difference in creatinine levels among the groups.

**Table 7:** Liver and kidney biochemical analyses of the untreated, negative control and aqueous and ethanolic extract groups during acute toxicity experiments

Parameter	Untreated	Negative	Aqueous extract	Ethanolic extract
Liver function tests				
AST (U/L)	143.50 ± 6.36	164.66 ± 3.33^a^	161.70 ± 8.30^a^	238.50 ± 2.50^a^
ALT (U/L)	33.25 ± 4.45	28.44 ± 2.98	27.70 ± 3.70	42.00 ± 4.35^a,b,c^
ALP (U/L)	84.50 ± 0.70	86.00 ± 3.05	80.55 ± 8.45	111.00 ± 7.02^a,b,c^
Kidney function tests				
BUN (mg/dL)	23.55 ± 0.21	24.00 ± 2.00	26.33 ± 2.08	31.00 ± 0.30^a,b,c^
Creatinine (mg/dL)	0.55 ± 0.06	0.56 ± 0.02	0.55 ± 0.01	0.54 ± 0.02

Data are the mean ± SD (*n* = 5 per group)

^a^*p* < 0.05, significantly different compared to the untreated group

^b^*p* < 0.05, significantly different compared to the negative control group

^c^*p* < 0.05, significantly different compared to the aqueous extract group

#### Histopathological analysis of the liver and kidneys

**Fig. 1:**
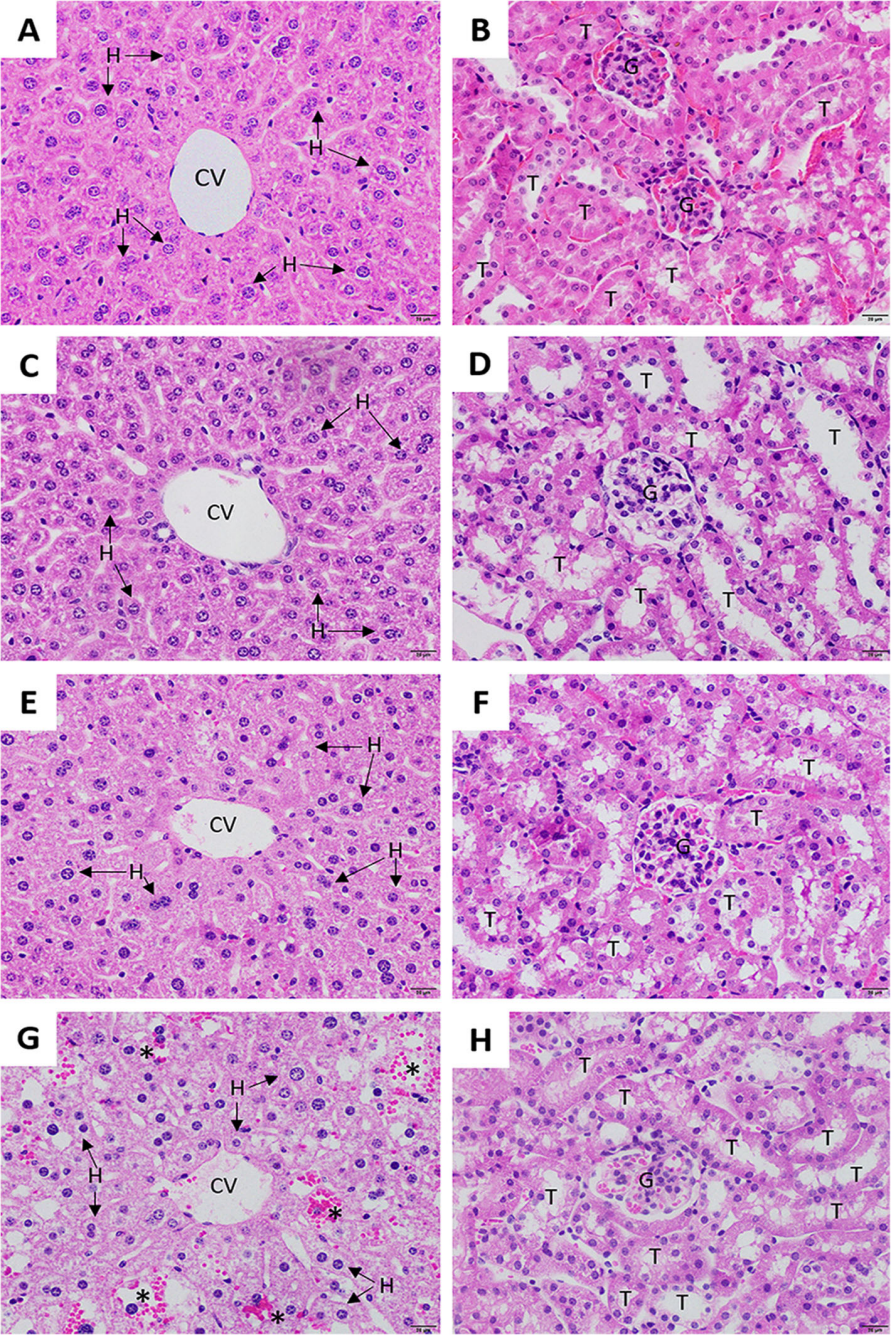
Histopathology of the liver and kidney of mice during the acute toxicity test of the untreated group (**a** and **b**), the negative control group (**c** and **d**), the group treated with 2000 mg/kg aqueous *S. exigua* root extract (**e** and **f**) and the group treated with 2000 mg/kg ethanolic *S. exigua* root extract (**g** and **h**). H hepatocyte, CV central vein, S sinusoidal capillary, G glomerulus, T tubule. The asterisk shows the area of hepatic hemorrhage

The histological architecture of the liver in the untreated and negative control groups showed normal morphology of hepatocytes with an acidophilic cytoplasm and normal structure of hepatic sinusoids and the central vein (Fig. 1). The hepatological analysis of mice treated with aqueous extract at a single dose of 2000 mg/kg showed mild hepatocyte necrosis and infiltration of inflammatory cells. Mice treated with ethanolic extract at a single dose of 2000 mg/kg showed ballooning degeneration of hepatocytes, diffuse hepatic hemorrhage and infiltration of inflammatory cells. The histopathological assessment of the kidney in all groups showed no change in the histological architecture of glomerular and renal tubules compared to the untreated group.

## Discussion

In this study, the antioxidant properties of aqueous and ethanolic *S. exigua* root extracts were determined by the DPPH radical assay and SRSA and HRSA assays. The three antioxidant assays are commonly used for screening the in vitro antioxidant properties of plant extracts [21, 22]. Our study showed that the ethanolic extract of *S. exigua* root exhibited inhibition of DPPH radicals, superoxide anions and hydroxyl radicals, with IC_50_ values of 24.63 ± 1.78, 129.78 ± 0.65 and 30.58 ±1.19 μg/ml, respectively. A DPPH radical assay of plant ingredients in Kheaw-hom remedy showed that the ethanolic extract of *S. exigua* root strongly inhibited DPPH radicals with an IC_50_ value of 9.42 ± 2.11 μg/ml [12], which was similar to the result of this study. The results from phytochemical screening of the *S. exigua* root extract showed that the ethanolic extract contained flavonoids, including terpenoids, saponins and coumarins, whereas the aqueous extract contained alkaloids, flavonoids and terpenoids. These results are consistent with a previous report showing that the *Sophora* genus contains four main groups, namely, alkaloids, flavonoids, polysaccharides and fatty acids [11]. A previous study reported that plant crude extracts containing flavonoids and polyphenol compounds act as reducing agents and antioxidants [23]. These compounds contain hydroxyl groups and mediate their antioxidant effects by scavenging free radicals [24]. They act as hydrogen-donating antioxidants and can react with lipid peroxyl radicals, resulting in breakage of the generation cycle of new radicals [25].

To evaluate the in vivo antioxidant properties of *S. exigua* root extract, we demonstrated that malaria-infected mice treated with ethanolic extract at doses of 200, 400 and 600 mg/kg had significantly reduced MDA levels compared to the infected untreated group. The mean rate of SOD inhibition was higher in the tissue homogenate of malaria-infected mice treated with ethanolic and aqueous extracts at doses of 200, 400 and 600 mg/kg than in tissue homogenates from the infected untreated group. The MDA level and SOD activity are commonly used as measures of oxidative stress in cardiovascular disease [26] and malaria infection [27]. During plasmodium parasite infection, increased MDA levels and decreased SOD activity were found in human and murine models [28, 29]. Sophoraflavanone G extracted from *S. exigua* roots exerts antimicrobial activity by decreasing the fluidity of cellular membranes [14]. Additionally, it has been reported that sophoraflavanone treatment led to increased SOD activity and decreased MDA levels in the serum of diabetic rats with streptozotocin (STZ)-induced inflammation [30].

According to the 4-day suppressive test, the crude extracts were found to decrease the level of parasitemia in a dose-dependent manner. This study demonstrated that the aqueous extract of *S. exigua* root exhibited stronger antimalarial activity than the ethanolic extract. The aqueous extract at a dose of 600 mg/kg suppressed parasitemia with a suppression value of 60.46%. According to the results of this study, this effect may be attributed mainly to the alkaloid, flavonoid and terpenoid compounds that were the secondary metabolites present in the aqueous extract. Regarding alkaloids, several classes of these compounds, including indole, bisindole, quinolone and isoquinoline alkaloids isolated from different medicinal plants, were identified as having promising antimalarial activity [31]. A previous study reported that some sesquiterpenoid and triterpenoid compounds inhibit parasite growth by inhibiting the polymerization of heme to hemozoin through free radical formation of sesquiterpenic lactone. Furthermore, these compounds were reported to inhibit protein synthesis in parasitic cells and inhibit *Pf*ATP6, a Ca^2+^ ion transporter that is a sarcoendoplasmic reticulum calcium-dependent ATPase (SERCA) [32]. Additionally, monoterpenoid indole alkaloids such as uleine also inhibit heme polymerization as a result of the presence of a basic aliphatic amino group that undergoes protonation in the acidic digestive vacuole [33]. For flavonoids, it is believed that this compound acts by inhibiting fatty acid biosynthesis (FAS II) in the parasite. In addition, some flavonoids have also been reported to inhibit the growth of malaria parasites by inhibiting the influx of l-glutamine and myoinositol into infected erythrocytes [34]. Therefore, antimalarial effect of aqueous extract of *S. exigua* root against *P. berghei* ANKA might be attributed to a single or a combination of its secondary metabolites, leading to alteration of protein structure and impairment of its function and inhibition of blood stage propagation of parasites [35].

A previous study showed that an isolated *S. exigua* root extract contained exiguaflavanone A, exiguaflavanone B and sophoraflavanone G [36]. It has been reported that the flavanone compounds exiguaflavanones A and B isolated from *Artemisia indica* Willd exhibited in vitro antimalarial activity against *P. falciparum* with IC_50_ values of 4.60 and 7.05 μg/ml, respectively [37]. Antimalarial testing of (2*S*)-2′-methoxykurarinone, sophoraflavanone G and leachianone A isolated from the roots of *S. flavescens* demonstrated moderate antimalarial activity, with IC_50_ values of 2.4, 2.6 and 2.1 μM, respectively [10]. The effect of *S. exigua* on antimalarial activity may be due to exiguaflavanones A and B as well as sophoraflavanone G, which might be the active compounds in this plant. Therefore, our study suggests that further investigation is required to characterize the active compounds of the aqueous extract of *S. exigua* roots that have potent effects on the inhibition of malaria parasites.

Regarding acute oral toxicity, the ethanolic and aqueous extracts did not cause any obvious mortality in the experimental mice at 2000 mg/kg oral administration. This revealed that a lethal dose of 50% is greater than 2000 mg/kg. In the present study, the mice were observed daily for 14 days to determine body weight changes, and changes in water and food consumption. The percentage of body weight change was very less in those mice treated with both crude extracts as compared with mice in untreated group. The water intake and food consumption in mice that received ethanolic extract were significantly reduced. Therefore, ethanolic extract might be affect increasing body weight through appetite suppression.

In this study, the plasma levels of AST, ALT, ALP and BUN were increased in the mice treated with 2000 mg/kg ethanolic extract compared to the untreated group. This finding was related to the results obtained from histopathological analysis of liver tissue. The histopathological analysis revealed that mice treated with ethanolic extract at a single dose of 2000 mg/kg showed ballooning degeneration of hepatocytes, diffuse hepatic hemorrhage, and infiltration of inflammatory cells. The liver is a primary organ involved in the biotransformation of food and drugs after oral administration. The cause of hepatic injury is toxic chemicals; xenobiotics; anticancer, immunosuppressant, analgesic, anti-inflammatory, and antitubercular drugs; and biological agents that show the ability to induce hepatic injury [38]. Previous studies have demonstrated that sophoraflavanone G isolated from *S. exigua* root and *S. flavescens* has cytotoxic effects on human myeloid leukemia HL60 cells [39, 40]. In addition, it has been reported that sophoraflavanone G has potent toxic effects on primary rat hepatocytes and human HL-7702 liver cells, with IC_50_ values of 16.5 and 40.3 μM, respectively [41]. A previous study showed that sophoraflavanone G inhibited cytochrome P450 activity by inhibiting CYP2B6 and CYP3A4 [42]. Therefore, the ethanolic extract of *S. exigua* roots is required for further investigation to confirm its safety at lower doses.

## Conclusions

This study demonstrated that the ethanolic *S. exigua* root extract possesses antioxidant properties and that the aqueous extract also has antimalarial activity. Additionally, hepatotoxic effects of the *S. exigua* root ethanolic extract were observed at 2000 mg/kg body weight. The appropriate dose of *S. exigua* root extract for antimalarial activity needs to be further investigated.

## Data Availability

All data generated or analyzed during this study are available from the corresponding author on reasonable request.
